# Supplementary material for: HIVprotI: an integrated web based platform for prediction and design of HIV proteins inhibitors
Source: J Cheminform. 2018 Mar 9;10:12. doi: 10.1186/s13321-018-0266-y (PMC5845081; doi:10.1186/s13321-018-0266-y)
Supplement: Supplementary file 2 — Additional file 2. Source code of HIVProtI web server. [file 13321_2018_266_MOESM2_ESM.zip › HIVprotI_Source-code/hivprotI/weka-3-6-6/doc/weka/classifiers/package-frame.html]

weka.classifiers


weka.classifiers

Interfaces 
  
*IntervalEstimator*
  
*IterativeClassifier*
  
*Sourcable*
  
*UpdateableClassifier*

Classes 
  
BVDecompose
  
BVDecomposeSegCVSub
  
CheckClassifier
  
CheckSource
  
Classifier
  
CostMatrix
  
Evaluation
  
IteratedSingleClassifierEnhancer
  
MultipleClassifiersCombiner
  
RandomizableClassifier
  
RandomizableIteratedSingleClassifierEnhancer
  
RandomizableMultipleClassifiersCombiner
  
RandomizableSingleClassifierEnhancer
  
SingleClassifierEnhancer
